# Rhinitis, sinusitis and ocular disease – 2102. The activities of allergy pot as a patient group in Japan: developing treatment guidelines

**DOI:** 10.1186/1939-4551-6-S1-P176

**Published:** 2013-04-23

**Authors:** Mariko Kuriyama, Mari Shiozaki

**Affiliations:** 1 Allergy Pot: Network Supporting Children with Allergy Certified Non-Profit Organization, Japan

## 

Among the 120 million people living in Japan, it is estimated that approximately half of the population is suffering from some kind of allergy, and that there are about 7 million adults and children who are suffering from asthma alone.

“Allergy Pot” is a parent group founded in 2002, with the prospect of “disseminating information for a better understanding of allergy in educational institutions by respecting their situation and working together, to build a basis for a safe educational environment." We started out from making “Going to School ” booklets, to help the schools and classmates have a better understanding of allergy. We planned, produced, and distributed these booklets, and also made it downloadable from our website.

Searching through the internet using the key words “allergy patient group” from either Google or Yahoo websites (from 300 results) shows that there are about 40 patient groups related to allergic diseases in Japan. Most of the allergy patient groups in Japan are self-help groups. Allergy Pot is unique in the sense that it is a group that is continuously sending out messages from the standpoint of patients and patient supporters with the vision and policy that “patient groups are social resources, and our goal is to play our role and contribute in society.” We have gained special recognition by the educational institutions, academic society, administration and citizens for: (1)participating in the process of developing treatment guidelines, (2)being involved not only in allergy related meetings, but numerous administrative meetings related to medical policy making, and for (3)working in cooperation with the Tohoku Medical Megabank Organization to support the reconstruction of Eastern Japan after the earthquake.

The history and specific activities of our patient group will be introduced, along with how we have been able to build up a cooperative relationship with the medical and governmental fields, and how we were able to have an influence in the medical administration in Japan. We would also like to discuss the role of patient groups and its possibilities in a country such as Japan, where every citizen is insured under the national health insurance system.

